# Avoided Heat-Related Mortality through Climate Adaptation Strategies in Three US Cities

**DOI:** 10.1371/journal.pone.0100852

**Published:** 2014-06-25

**Authors:** Brian Stone, Jason Vargo, Peng Liu, Dana Habeeb, Anthony DeLucia, Marcus Trail, Yongtao Hu, Armistead Russell

**Affiliations:** 1 School of City and Regional Planning, Georgia Institute of Technology, Atlanta, Georgia, United States of America; 2 Center for Sustainability and the Global Environment, University of Wisconsin-Madison, Madison, Wisconsin, United States of America; 3 School of Civil and Environmental Engineering, Georgia Institute of Technology, Atlanta, Georgia, United States of America; 4 Quillen College of Medicine, East Tennessee State University, Johnson City, Tennessee, United States of America; US Army Engineer Research and Development Center, United States of America

## Abstract

Heat-related mortality in US cities is expected to more than double by the mid-to-late 21^st^ century. Rising heat exposure in cities is projected to result from: 1) climate forcings from changing global atmospheric composition; and 2) local land surface characteristics responsible for the urban heat island effect. The extent to which heat management strategies designed to lessen the urban heat island effect could offset future heat-related mortality remains unexplored in the literature. Using coupled global and regional climate models with a human health effects model, we estimate changes in the number of heat-related deaths in 2050 resulting from modifications to vegetative cover and surface albedo across three climatically and demographically diverse US metropolitan areas: Atlanta, Georgia, Philadelphia, Pennsylvania, and Phoenix, Arizona. Employing separate health impact functions for average warm season and heat wave conditions in 2050, we find combinations of vegetation and albedo enhancement to offset projected increases in heat-related mortality by 40 to 99% across the three metropolitan regions. These results demonstrate the potential for extensive land surface changes in cities to provide adaptive benefits to urban populations at risk for rising heat exposure with climate change.

## Introduction

Human health effects associated with rising temperatures are expected to increase significantly by mid-to-late century. A large body of work now estimates an increase in mean global temperature from pre-industrial averages of more than 2°C by late century under mid-range emissions scenarios [1]. A smaller but growing body of work has sought to estimate the effects of projected warming on heat-related mortality. Employing health impact functions derived from epidemiological studies of historical warm season mortality rates, recent work projects an increase in annual heat-related mortality of between 3,500 and 27,000 deaths in the United States by mid-century [2]. Studies focused on individual cities estimate an increase in annual heat-related mortality by a factor of 2 to 7 by the mid-to-late 21^st^ century [3]–[5].

The urban heat island effect compounds the potential effects of global scale climate change on heat-related mortality among urban populations. Time series analyses of climatic trends in cities find large urbanized regions to be warming at a higher rate than proximate rural areas, with many cities warming at more than twice the mean global rate [6], [7]. The combined effects of urban heat island formation and the global greenhouse effect are projected to significantly increase the number of extreme heat events in urbanized regions [8]. At present, the extent to which the urban heat island effect may further increase heat-related mortality is not well established.

Here we examine the potential for urban heat island mitigation as a climate adaptation strategy to reduce projected heat-related mortality in three large US cities by mid-century. Future year climate and seasonal mortality are modeled across the metropolitan statistical areas (MSAs) of Atlanta, Georgia, Philadelphia, Pennsylvania, and Phoenix, Arizona to capture a wide continuum of climatic, geographic, and demographic characteristics known to underlie population vulnerability to extreme heat. Using coupled global and regional scale climate models together with an environmental health effects model, we project the number of heat-related deaths expected for these regions in 2050 in response to a “business as usual” (BAU) and an array of urban heat management scenarios characterized by variable land cover modifications. Employing separate health impact functions responsive to temperature change and derived from prior epidemiological studies, referred to herein as “heat response functions” (HRFs), we find different combinations of heat management strategies to offset projected increases in heat-related mortality across the three MSAs by a range of 40 to 99%.

Our work builds on previous studies of climate change and heat-related mortality in three respects. First, we develop a set of climate projections responsive not only to future changes in atmospheric composition but to changes in land cover characteristics as well to capture the influence of heat island formation on heat-related health outcomes. Second, in addition to estimating changes in heat-related mortality resulting from future year climatic conditions, we further model the influence of alternative heat management strategies on health outcomes. Third, we introduce a modeling approach that enables health outcomes resulting from mean warm season temperatures and shorter-term heat wave events to be estimated by employing multiple HRFs.

## Methods

### Land cover modeling

The influence of local climate modification on heat-related mortality was estimated through the integration of separate land cover, climate, and human health effects models. To account for separate global and regional climate forcings on future climate, our approach made use of a land cover modeling routine responsive to historical rates of land cover change. As presented in an earlier paper [9], historical land cover change rates by urbanization class were developed for each metropolitan region from the National Land Cover Database [10] and projected forward based on population projections for each decade from 2010 to 2050.

Employing this approach, the type and area of vegetative (tree canopy, grass, shrubland, agriculture) and impervious (building roofing, street paving, other surface paving) land cover were estimated across the three metropolitan regions for a 2050 BAU scenario linking land cover change to population growth at the census tract level. Under the BAU scenario, historical rates of land cover change per unit of population growth, such as the area of forestland lost with each new 10,000 residents added to a census tract, are expected to continue into the future. Based on projected population growth per census tract between 2010 and 2050, we estimated how seven classes of land cover, including forest, grass, barren land, water, wetlands, agriculture, and impervious surfaces, are expected to change by 2050. These tract-level land cover values were then aggregated to a 4 km model grid resolution and used as land surface inputs to a mesoscale meteorological model.

**Figure 1:**
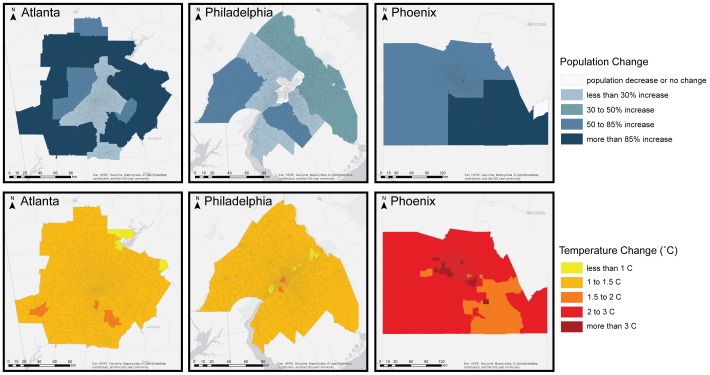
Change in population and average warm season (May – Sept) temperature under the BAU scenario between 2010 and 2050.

County level population data were obtained for all counties in the Atlanta, Philadelphia, and Phoenix MSAs from the economic forecasting firm *Woods & Poole* for the years 2010 to 2040 and then extrapolated to 2050 with a statistical routine employing ordinary least squares. County level population estimates stratified by age cohorts and race/ethnicity were then disaggregated to the census tract level with a spatial weighting algorithm. Figure 1 (top panel) illustrates the percent change in population between 2010 and 2050 in each MSA. These maps project Atlanta and Phoenix to grow more rapidly than Philadelphia, and for expected growth to continue to largely occur in suburban zones. The population projections used in this study are publicly available through the following URL: http://doi.org/10.5061/dryad.14g40.

### Global and regional climate modeling

Future year climatic conditions were simulated though the coupling of the Weather Research Forecasting (WRF) mesoscale meteorological model to the Goddard Institute for Space Studies (GISS) Global Atmosphere-Ocean ModelE (the GISS-WRF model system) [11]–[13]. The coarse resolution (2°×2.5°) meteorological fields of the GISS ModelE global circulation model (GCM) were dynamically downscaled to 36 km, after which each metropolitan study region was nested and downscaled to 12 and 4 km to capture fine scale land use impacts and temperature variations. Energetic parameters in WRF were set to correspond with either base year (2010) or projected 2050 land cover attributes in each region, aggregated to the 4 km grid resolution. Simulated meteorological fields of interest included hourly ambient temperature (2 m), surface skin temperature, ambient humidity (2 m), surface heat fluxes (latent, sensible, and radiation), precipitation, wind velocities, and turbulence.

The GCM projections used for this study were obtained from a previous study by Trail and others [14]. Through this previous study, the performance of the GISS model in reproducing observed warm season temperatures across the continental US over the period of 2006 to 2010 was assessed and found to have a moderate cool bias of −1.9°C, with regional mean biases of −0.2°C, −2.0°C, and −4.2°C, in the south, northeast, and west, respectively. These results compare favorably with other studies making use of the GISS model [15], [16].

Meteorological fields corresponding to one emissions and eight land cover scenarios were simulated through WRF for the full year of 2050. In order to assess the extent to which modeled temperature changes fall beyond the range of natural variability captured in the WRF model, we generated a five-member ensemble of the BAU scenario by modifying the initial conditions in each simulation. The results of this ensemble modeling yielded standard errors averaging 0.112°C across the three temperature metrics in Atlanta, 0.018°C in Philadelphia, and 0.004°C in Phoenix. Supplemental Figure S1 and Table S1 summarize in more detail the results of these ensemble simulations.

While it is a conventional practice to average future year climate estimates over successive years, we made use of a single year of simulated meteorology in this study to capture heat wave episodes that may have been moderated or lost from a statistical smoothing of temperature projections across multiple years. A comparison of GISS model results across the five years of 2048 through 2052 did not find 2050 to be a statistically anomalous year for any of the meteorological fields of interest [14]. In each case, the Intergovernmental Panel on Climate Change (IPCC) RCP4.5 emissions scenario was used for the GISS model runs, corresponding to “middle of the road” emissions assumptions. As our interest in this study is on the influence of alternative land cover change scenarios on future regional climates, rather than on the influence of alternative global emissions scenarios, we make use of a single emissions forecast and then assess how temperature and heat-related mortality vary in response to changing land cover conditions at the metropolitan scale. The RCP4.5 emissions scenario was selected for the GCM projection as it represents middle range assumptions pertaining to emissions growth and radiative forcing.

The influence of alternative heat management scenarios was assessed through separate WRF runs parameterized around variable land cover assumptions. These WRF runs included two scenarios focused on vegetation enhancement differentiated by property type (private vs. public land parcels), two scenarios focused on albedo enhancement differentiated by material type (roofing vs. surface paving), and three scenarios employing varying combinations of these heat management strategies. Table 1 presents a detailed overview of each heat management scenario included in the study. All WRF model output is publicly available through the following URL: http://doi.org/10.5061/dryad.14g40.

**Table 1:** Description of 2050 heat management WRF simulations.

Scenario definition	Type of land cover modified	Type of modification
Private Greening (PRG)		
*trees, grass, and/or shrubs added to private property to achieve minimum 80% green cover for residential parcels and minimum 50% green cover for non-residential parcels*	*building roof and surface paving (e.g., driveways and sidewalks) on private parcels*	*1. All commercial roofs converted to grass. 2. Surface paving and building roofs overlaid with tree canopy or converted to grass/shrubs to achieve green area minimum by land use class*
Public Greening (PUG)		
*trees, grass, and/or shrubs added to public property to achieve or approach green area minimum of 80% for publicly owned land*	*street surfaces, parkland, and other publicly owned parcels*	*1. 50% of all roadway surfaces overlaid by tree canopy. 2. Grass or barren land in public parcels converted to tree canopy (ATL and PHL). 3. Barren or agricultural land in public parcels converted to a grass/shrub mix (PHX).*
Building Albedo Enhancement (BAE)		
*increase the albedo of building roof surfaces*	*all building roofs*	*converted to high albedo (0.9) impervious surfaces*
Road Albedo Enhancement (RAE)		
*increase the albedo of paved surfaces*	*all roads, parking lots, and other surface paving*	*converted to moderate albedo (0.45) impervious surfaces*
Combined Green Strategies (GREEN)		
*combination of PRG and PUG scenarios*	see PRG and PUG scenarios	see PRG and PUG scenarios
Combined Albedo Strategies (ALBEDO)		
*combination of BAE and RAE scenarios*	*see BAE and RAE scenarios*	*see BAE and RAE scenarios*
All Strategies Combined (ALL)		
*combination of PRG, PUG, BAE, RAE scenarios*	*see PRG, PUG, BAE, and RAE scenarios*	*for areas subject to either vegetation or albedo enhancement, vegetation enhancement is prioritized*

To date, numerous observational and modeling studies have found vegetative cover and high albedo roofing and paving materials – referred to generally as “cool materials” – to be associated with lower surface and near-surface air temperatures than sparsely vegetated areas with low albedo impervious materials [17]–[19]. In some studies the cooling benefits of vegetative and high albedo materials are shown to extend beyond the zones in which these materials are found [20]. The climatic benefits of these land cover types result from either an increase in local rates of evapotranspiration, which offsets sensible heating at the surface, or through an increase in shortwave reflection, reducing the absorption of solar energy [21]. The heat management strategies outlined in Table 1 are designed to increase vegetative and high albedo materials separately and in combination to assess the relative impacts of each type of intervention, as well as to establish feasible land cover change goals achievable through municipal land use policies. Consistent with environmental zoning policies recently adopted in Seattle, WA and Washington, DC [22], several of our scenarios set minimum green area targets per parcel to bring about land cover changes associated with urban heat island mitigation.

**Figure 2:**
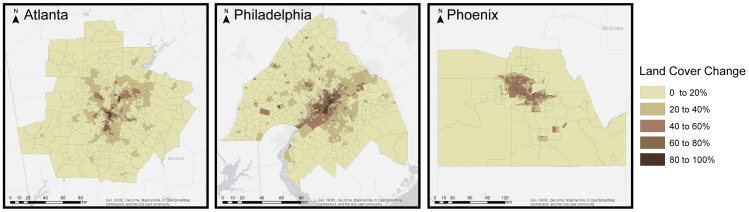
Percent of census tract area modified through the ALL heat management scenario. Land cover changes include the addition of new tree canopy, grass, or shrubs; conversion of roofing areas to greenroofs or high albedo materials; and the conversion of streets and other surface paving to moderate albedo materials (see Table 1).

To assess the potential for municipal governments to reduce urban temperatures through strategies focused on publicly managed land alone, we further stratify our vegetation and albedo enhancement scenarios by land ownership class. As such, the Public Greening (PUG) and Road Albedo Enhancement (RAE) scenarios modify the land cover characteristics of publicly owned street surfaces, parks, and other publicly owned parcels, while the Private Greening (PRG) and Building Albedo Enhancement (BAE) scenarios modify the land cover characteristics of privately owned parcels across each region. The combined scenarios of GREEN, ALBEDO, and ALL enable the assessment of vegetation and albedo enhancement alone or in combination across all land ownership types. Figure 2 illustrates the percent area of each census tract modified through the heat management strategies included in the ALL scenario, showing a concentration of these strategies in the highest density tracts where impervious cover is greatest.

### Health effects modeling

The implications of each land cover change scenario for heat-related mortality were assessed with an environmental health effects model developed by the US Environmental Protection Agency, the *Environmental Benefits Mapping and Analysis Program* (BenMAP). Constructed as a damage function estimation tool, the BenMAP model estimates excess mortality resulting from heat exposure through the following function:
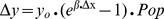
where Δy is the estimated change in mortality, y_o_ is the baseline incidence rate for a specified cause of mortality, β is a measure of relative risk obtained from surveillance studies of heat mortality, Δx is the estimated change in heat exposure, and Pop is the size of the exposed population.

Initially developed for the purpose of estimating health benefits associated with air pollution control strategies, the BenMAP model has recently been adapted to treat heat exposure as a pollutant through the integration of published impact functions for temperature [2]. In this study, we employ an approach similar to that developed by Voorhees and others [2], which estimated national excess heat-related mortality associated with projected global temperature change between the present period and 2050. The BenMAP model offers two important benefits to our study. First, its use for the estimation of climate change-related health effects has been validated through an initial “proof of concept” paper focused on the same period of projection. Second, the BenMAP model provides a standardized set of present-day and future year estimates of baseline incidence (y_o_ – the number of deaths expected to occur independent of changes in temperature) for all causes of mortality at the county level across the US. As such, use of the BenMAP model for climate and health research enhances the comparability of different studies focused on the health risks of temperature change over time.

We selected from the epidemiology literature three published measures of relative risk (β) quantifying excess mortality above baseline incidence rates per unit change in temperature or per heat wave day. Separate HRFs for average warm season mortality were modeled to account for differing temperature-mortality associations established in response to alternative temperature metrics. As presented in Table 2, HRFs developed by Medina-Ramon and Schwartz [23] and Zanobetti and Schwartz [24] are responsive to differences between scenarios in either average daily minimum temperatures (“Medina-Ramon HRF”) or average daily mean apparent temperatures (“Zanobetti HRF”) over a May through September warm season.

**Table 2:** Description of heat response functions.

Study	Temperature metric	Relative risk	Mortality type	Study population
Anderson and Bell, 2011	heat wave periods classified as 2 or more days with mean daily T above 95th percentile of 1987–2005 average for May-Sept	1.0367 (1.0295, 1.0439) per heat wave day	non-accidental	all ages in 43 US cities (1987–2005)
Medina-Ramon and Schwartz, 2007	minimum daily T (May-Sept) above 17°C; measured as 2-day cumulative T	1.0043 (1.0024, 1.0061) per 1°C (O_3_ adjusted)	all cause	all ages in 42 US cities (1989–2000)
Zanobetti and Schwartz, 2008	mean daily apparent temperature (May-Sept)	1.018 (1.0109, 1.025) per 5.55°C (O_3_ and PM_2.5_ adjusted)	non-accidental	all ages in 9 US cities (1999–2002)

Confidence intervals (95%) for estimates of relative risk in parentheses.

Prior work has found HRFs constructed from seasonal or annual health surveillance data to underestimate heat-related mortality during episodic heat wave conditions [25], [26]. To capture the additive effect of heat wave conditions on mortality rates, we make use of a secondary HRF developed by Anderson and Bell [26], which measures the additional risk of heat exposure during consecutive days of extreme temperatures. The “Anderson HRF” measures the increase in mortality per heat wave day, defined as one of two or more consecutive days in which the mean temperature exceeds the 95^th^ percentile of the long-term (1987–2005) daily mean temperature for each MSA. Mean temperature thresholds of 28.7°C, 28.8°C, and 37.3°C were used to identify heat wave periods in Atlanta, Philadelphia, and Phoenix, respectively (temperature thresholds provided by Dr. Brooke Anderson, December 2013).

## Results

Temperature change was first modeled for the 2050 BAU scenario in reference to base year (2010) conditions in each metropolitan region. Three measures of temperature change were derived for each MSA to correspond with the HRFs used to assess health outcomes, including average temperature (AvgT), average apparent temperature (AvgapT), and minimum temperature (MinT). Under the BAU scenario, warm season temperatures increase from base year temperatures by an average of 1.2°C in the Atlanta and Philadelphia MSAs, and by an average 2.2°C in the Phoenix MSA (see Figure 1, bottom panel).

**Figure 3:**
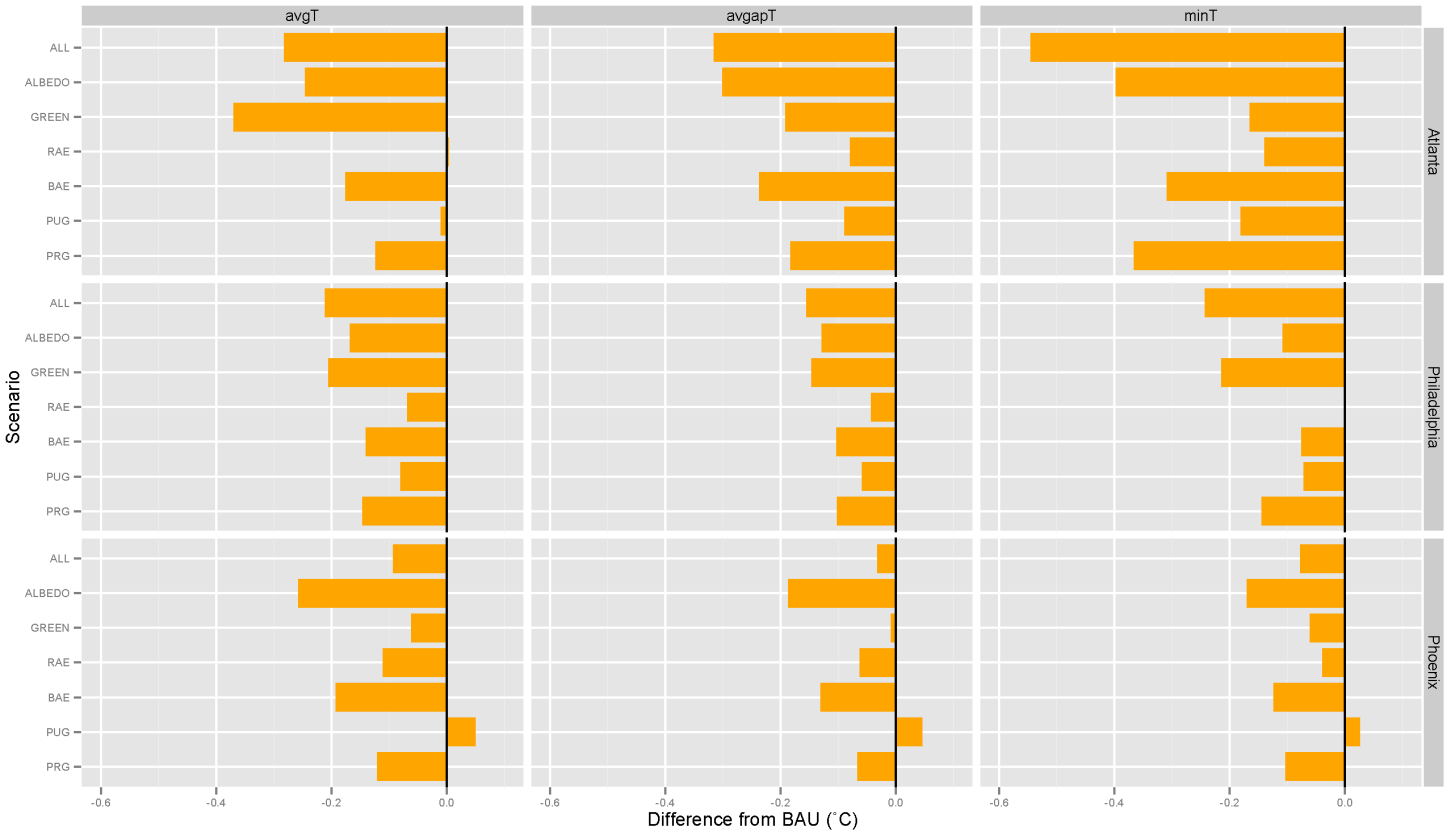
Differences in warm season temperature from BAU by heat management scenario, temperature metric, and MSA.

Figure 3 presents the results of the heat management scenario modeling relative to BAU conditions. Across the MSA-temperature metric combinations, the influence of variable heat management strategies on BAU temperatures was found to range from an increase in mean warm season apparent temperature of 0.06°C to a reduction in minimum temperature of 0.57°C. In Atlanta and Philadelphia, maximum temperature reductions were achieved through either vegetation enhancement or a combination of vegetation and albedo enhancement. By contrast, albedo enhancement alone was associated with the greatest temperature reductions in Phoenix. Fifty-eight of the 63 scenarios presented in Figure 3 were associated with reductions in average MSA temperatures relative to BAU during the warm season. In addition, the multi-strategy ALL, ALBEDO, and GREEN scenarios were found to produce a cooling effect beyond the uncertainty ranges estimated through the ensemble modeling discussed above (Figure S1 and Table S1).

The variable effectiveness of the heat management strategies in lowering metro-wide, average warm season temperatures is driven in large part by differences in the area of land conversions and rates of soil moisture availability. In the eastern US, where annual precipitation rates are high, both the spatial extent and species mix of vegetation are conducive to higher rates of evapotranspiration than found in the arid climate of Phoenix, promoting in these areas more regional cooling through the latent heat flux. In Atlanta and Phoenix, albedo enhancement, on average, is found to be more effective than the combined green strategies due to the fact that more land area is available for modification. In these cities, the total area of land converted to high albedo materials is about one-third greater than that total land area subject to vegetation enhancement, yielding a greater cooling effect in most scenario/temperature metric combinations.

**Figure 4:**
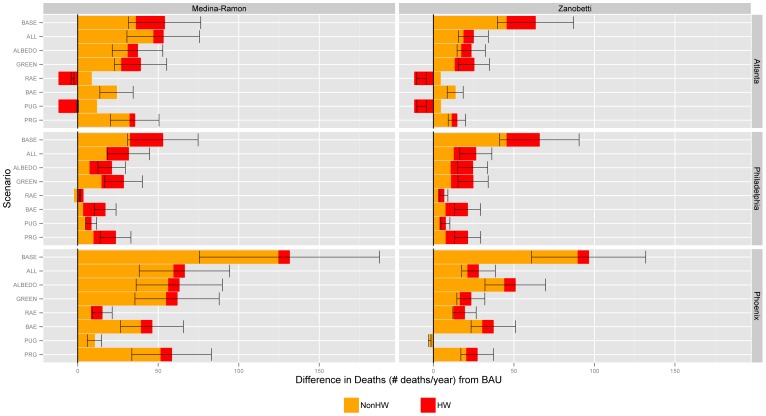
Difference in mortality relative to BAU by heat management scenario, HRF, and MSA. Bars report estimated difference in mortality relative to BAU in response to either the Medina-Ramon minimum temperature (minT) or Zanobetti average apparent temperature (avgapT) HRFs (orange shading) combined with the Anderson average temperature (avgT) HRF for heat wave conditions (red shading). Positive results denote a reduction in mortality relative to the BAU scenario; negative results denote an increase in mortality relative to the BAU scenario. Error bars report 95% confidence intervals.

Consistent with reported temperature change, trends in heat-related mortality are reported in Figure 4 as differences in the number of deaths relative to the BAU scenario (deaths under BAU scenario minus deaths under heat management scenario). The total number of avoided (or increased) deaths reported for each scenario accounts for non-heat wave and episodic heat wave periods during the 2050 warm season in each region. The “BASE” scenario reports the projected number of deaths resulting from changes in climate between the base year of 2010 and the 2050 BAU scenario, holding regional population characteristics constant at 2050 levels. The projected increase in annual heat-related mortality in response to warming over this 40-year period ranges from a low of 53 additional deaths in Philadelphia to a high of 132 additional deaths in Phoenix. Relative to base year levels, these 2050 projections represent an increase in heat-related mortality of 55% in Phoenix, 77% in Atlanta, and 319% in Philadelphia.

Heat management strategies offset projected increases in heat-related mortality within a 95% confidence level in 37 of 42 scenario runs. Mirroring the temperature change results, vegetation enhancement or a combination of vegetation and albedo enhancement resulted in the greatest reductions in BAU mortality in Atlanta and Philadelphia, while albedo enhancement in Phoenix was found to have the most significant effect on heat-related mortality in response to the Zanobetti/Anderson HRFs. The most effective heat management strategies in each region were found to offset projected increases in heat-related mortality by between a low of 40% in Atlanta and Philadelphia (Zanobetti/Anderson HRFs) to a high of 99% in Atlanta (Medina-Ramon/Anderson HRFs), with an average reduction across all MSA and HRF combinations of 57%. We find vegetation and albedo enhancement in the Atlanta region to almost fully offset the projected increase in heat-related mortality associated with changes in minimum temperature between 2010 and 2050.

**Figure 5:**
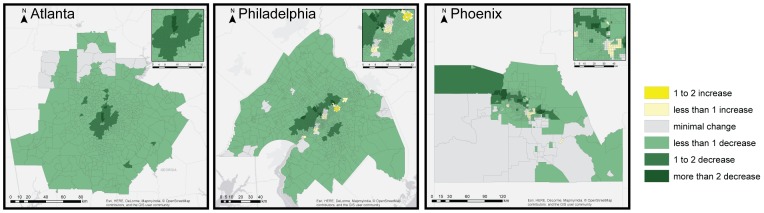
Change in mortality (per 100,000 population) under the ALL scenario in Atlanta, Philadelphia, and Phoenix. 2050 mortality changes are estimated in response to the Medina-Ramon/Anderson HRFs and are based on the difference in mortality between the BAU and ALL scenarios.

Figure 5 illustrates the change in heat-related mortality by census tract associated with the Medina-Ramon/Anderson HRFs, chosen due to evidence finding minimum temperatures to be the strongest predictor of heat-related health outcomes [27], [28]. The greatest concentration of avoided mortality is seen in the urban core of each metropolitan region, where population densities are high and the proportion of the land surface impacted by either albedo or vegetation enhancement is greatest. Heat-related mortality is shown to marginally increase in a small number of zones in the central districts of Philadelphia and Phoenix, an outcome attributed to the mixed thermal effects of tree planting along roadways and in public parks, as discussed below.

## Discussion

The results of our study support climate adaptation strategies designed to lessen the risk of heat exposure through mitigation of the urban heat island effect. Heat management strategies were found to be effective in offsetting mortality during both heat wave and non-heat wave conditions. Our results suggest that measures of relative risk for heat-related mortality based on average warm season temperatures only may significantly underestimate the potential for heat deaths during extreme heat events spanning two or more days. When accounting for both average warm season and heat wave conditions, the estimated number of avoided deaths due to the various heat management strategies was found to be 33% higher, on average, than model runs responsive to average warm season temperatures only.

The additional health benefits found to result from heat island mitigation during periods of extreme temperatures highlights the potential for established climate modeling protocols to systematically underestimate health risks associated with climate change. While a multi-year smoothing of climate projections may reduce the uncertainty of a median-year temperature estimate, it further carries the potential to obscure climate-related impacts associated with enhanced temperature variability. For this reason, standard climate modeling protocols that serve to statistically smooth annual and seasonal temperature variability may require modification when employed in health impact studies.

The heat management strategies most effective in offsetting mortality vary by region. Accounting for both warm season and heat wave deaths, vegetative strategies were found to have protective benefits greater than or comparable to albedo enhancement in Atlanta and Philadelphia, while albedo enhancement was found to be more protective in Phoenix. While the combined vegetation and albedo enhancement scenario (ALL) was generally found to be more effective in offsetting heat-related mortality, albedo strategies alone were found to be most protective of health in Phoenix in response to the apparent temperature response function (Zanobetti/Anderson HRF). The greater effectiveness of albedo strategies in arid climates reflects the limitations of vegetation enhancement in regions characterized by low soil moisture availability. The variable effect of heat management strategies by region demonstrates the need for heat abatement approaches to be tailored to the unique climatic conditions of different urban environments. Our findings further demonstrate the need to associate heat management strategies with a health endpoint directly, as some strategies found to be highly effective in reducing temperatures were less effective in offsetting heat-related mortality.

We find limited evidence that an increase in tree canopy on public property (PUG) in Atlanta and Phoenix may serve to marginally increase mortality – an effect that is not found when public tree planting is combined with other vegetation enhancement strategies. An examination of WRF model output for these scenario runs suggests the displacement of grass through tree planting may serve to elevate sensible heating through a dampening of the latent heat flux relative to BAU conditions. We conclude this outcome to result from an increase in leaf stomatal resistance with a shift from grass to tree canopy through this scenario, a physiological change that can decrease transpiration rates. Additionally, the PUG and RAE scenarios in Atlanta produce a larger number of heat wave days while simultaneously reducing average temperatures during heat wave episodes relative to BAU. As the heat wave response function is sensitive to changes in the number of heat wave days but not to changes in temperatures between scenarios, the modeled increase in heat wave mortality is in part an artifact of the heat wave response function employed in this study.

An additional limitation to our approach entails the use of historical temperature-mortality HRFs for the estimation of future year heat-related mortality [29]. If urban populations successfully exhibit physiological and/or behavioral (e.g., greater prevalence of air conditioning) adaptations to rising temperatures over time, the rate of mortality per degree rise in temperature or per additional heat wave day may be lower than reflected through the HRFs employed in this study.

## Conclusions

We examined the potential for urban heat management strategies to offset projected increases in heat-related mortality in three large US metropolitan regions by mid-century using a set of global/regional climate and human health effects models. Variable combinations of heat management strategies involving vegetation and albedo enhancement were estimated to offset projected heat-related mortality by a range of 40 to 99%, depending on the metropolitan region and health impact function applied. These results highlight the potential for extensive land surface changes in cities to provide adaptive benefits to urban populations at risk for rising heat exposure with climate change.

We believe the study findings can inform the development of urban heat adaptation plans through which municipal governments can moderate the extremity of ambient temperatures during heat wave events, in concert with the implementation of emergency operations plans designed to protect public health once such events are underway. In selecting among alternative heat management strategies, urban planners and public health officials will want to consider the extent to which vegetation and albedo enhancement are consistent with a range of other climate adaptation objectives – including stormwater and air quality management — as well as other stakeholder preferences [30]. Future work will estimate the air quality implications of the modeled land cover change in Atlanta, Philadelphia, and Phoenix, as well as the economic costs and benefits of the various heat management strategies evaluated herein.

## Supporting Information

Figure S1 **Uncertainty in WRF model initializations by temperature metric and MSA.** Error bars display confidence intervals around mean warm season temperatures (AvgT, AvgapT, and MinT) for five-member ensemble runs in which initial conditions were modified for the BAU scenario.
(TIF)

Table S1 **Mean warm season (May-Sept) standard errors (°C) from ensemble simulations by temperature metric and MSA.**
(DOCX)
