# Supplementary figures and images for: Avoided Heat-Related Mortality through Climate Adaptation Strategies in Three US Cities
Source: PLoS One. 2014 Jun 25;9(6):e100852. doi: 10.1371/journal.pone.0100852 (PMC4071007; doi:10.1371/journal.pone.0100852)

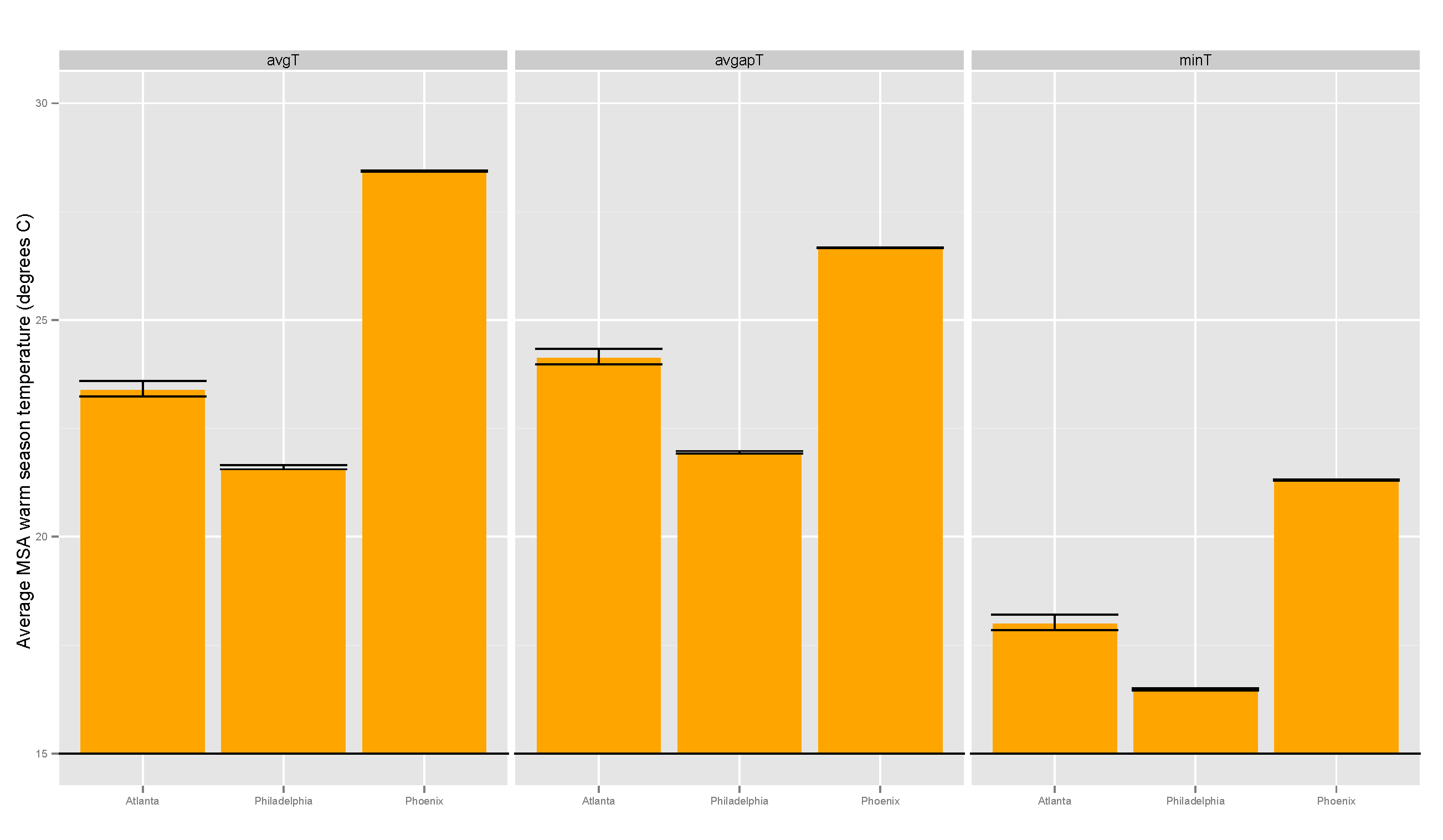

Supplement: Figure S1 — Uncertainty in WRF model initializations by temperature metric and MSA. Error bars display confidence intervals around mean warm season temperatures (AvgT, AvgapT, and MinT) for five-member ensemble runs in which initial conditions were modified for the BAU scenario. (TIF) [file pone.0100852.s001.tif]
