# Supplementary material for: Avoided Heat-Related Mortality through Climate Adaptation Strategies in Three US Cities
Source: PLoS One. 2014 Jun 25;9(6):e100852. doi: 10.1371/journal.pone.0100852 (PMC4071007; doi:10.1371/journal.pone.0100852)
Supplement: Table S1 — Mean warm season (May-Sept) standard errors (°C) from ensemble simulations by temperature metric and MSA. (DOCX) [file pone.0100852.s002.docx]

|  | **Atlanta** | **Philadelphia** | **Phoenix** |
| --- | --- | --- | --- |
| AvgT | 0.112 | 0.013 | 0.004 |
| AvgapT | 0.111 | 0.016 | 0.003 |
| MinT | 0.112 | 0.024 | 0.006 |
